# Maternal mortality and morbidity in the United States of America

**DOI:** 10.2471/BLT.14.148627

**Published:** 2015-03-01

**Authors:** Priya Agrawal

**Affiliations:** a Merck for Mothers, Merck & Co. Inc., 1 Merck Drive, Mail Stop WS2A-56, Whitehouse Station, New Jersey, 08889, United States of America.

Although considered mainly as problems of the developing world, maternal mortality and morbidity remain a challenge in the United States of America (USA).^1^ Between 1990 and 2013, the maternal mortality ratio for the USA more than doubled from an estimated 12 to 28 maternal deaths per 100 000 births^1^ and the country has now a higher ratio than those reported for most high-income countries and the Islamic Republic of Iran, Libya and Turkey.^2^ About half of all maternal deaths in the USA are preventable.^2^

Each year an estimated 1200^1^ women in the USA suffer complications during pregnancy or childbirth that prove fatal and 60 000^3^ suffer complications that are near-fatal – even though costs of maternity care in the USA in 2012 exceeded 60 billion United States dollars.^4^

Three factors are probably contributing to the upward trend in maternal mortality and morbidity in the USA. First, there is inconsistent obstetric practice. Hospitals across the USA lack a standard approach to managing obstetric emergencies and the complications of pregnancy and childbirth are often identified too late. Nationally endorsed plans to manage obstetric emergencies and updated training and guidance on implementing these plans is a serious and ongoing need.^5^

A second factor is the increasing number of women who present at antenatal clinics with chronic conditions, such as hypertension, diabetes and obesity, which contribute to pregnancy-related complications. Many of these women could benefit from the closer coordination of antenatal and primary care – including case management and other community-based services that help them access care and overcome cost and other obstacles. In the USA, women who lack health insurance are three to four times more likely to die of pregnancy-related complications than their insured counterparts.^6^

Another factor is the general lack of good data – and related analysis – on maternal health outcomes. Only half the USA’s states have maternal mortality review boards and the data that are collected are not systematically used to guide changes that could reduce maternal mortality and morbidity. There is no national forum for the states to share either their best practices for reviewing maternal deaths or the relevant lessons that they may have learned.

There is a growing effort by physicians, nurses and community organizations to address these three factors. Hospitals are beginning to implement standard approaches to managing obstetric emergencies so that, wherever a woman gives birth, she receives appropriate evidence-based care. Community initiatives are coordinating care for high-risk women to ensure good health and management of chronic conditions during and beyond pregnancy. More states are establishing or strengthening maternal mortality review boards.

Recent changes to national policies should also help improve maternal health outcomes. In 2010 the Affordable Care Act included antenatal and maternal care as essential health benefits that insurance plans must cover. By extending insurance coverage to pregnant women with low incomes, many states have lowered the economic hurdles that limit access to antenatal care for millions of women. As the health community solidifies the post-2015 agenda to end preventable maternal mortality, the USA needs to be brought into the global dialogue on maternal health. Although maternal mortality is relatively rare in the USA, one preventable maternal death is one too many. All states need to mobilize health providers, policy-makers and communities to make maternal health a priority. With increased awareness of maternal mortality and life-threatening events – and concrete actions to ensure that pregnant women get the quality care they need – many fatal and near-fatal complications could be prevented.
